# Patients colonization and infection in wards after discharge from a polyvalent intensive care unit with selective digestive decontamination: preliminary results

**DOI:** 10.1186/2197-425X-3-S1-A1014

**Published:** 2015-10-01

**Authors:** C Sánchez Ramirez, R Prada Osorio, CF Lübbe Vazquez, R Argandoña Primicia, J Cabrera Arrocha, F Artiles Campelo, S Ruiz Santana

**Affiliations:** University Hospital of Gran Canaria Dr Negrín, Intensive Care Unit, Las Palmas de Gran Canaria, Spain; University Hospital of Gran Canaria Dr Negrín, Microbiology Department, Las Palmas de Gran Canaria, Spain

## Objectives

To analyze the colonization and infection rate of patients after discharge from an the Intensive Care Unit (ICU) with Selective Digestive Decontamination (SDD).

## Methods

In a polyvalent ICU of 30 beds, from October 7^th^ to December 30^th^ 2014, SDD was applied to all patients requiring endotracheal intubation for more than 48 hours. We administered during the first four days intravenous cefotaxime plus enteral solution and a paste with colistin, tobramycin, and nystatin every 8 hours. Oropharyngeal, rectal and nasal swabs were obtained on admission, whether or not they received SDD and once weekly. To assess in the wards, after ICU discharge, colonization and development of hospital infections with germs originated in the ICU, pharyngeal and rectal swabs on the 3^th^ and 10^th^ day after ICU discharge were obtained and analyzed. Categorical variables were summarized as frequencies and percentages and number in means and standard deviations (SD) or median with interquartile ranges (IQR).

## Results

**Figure 1:**
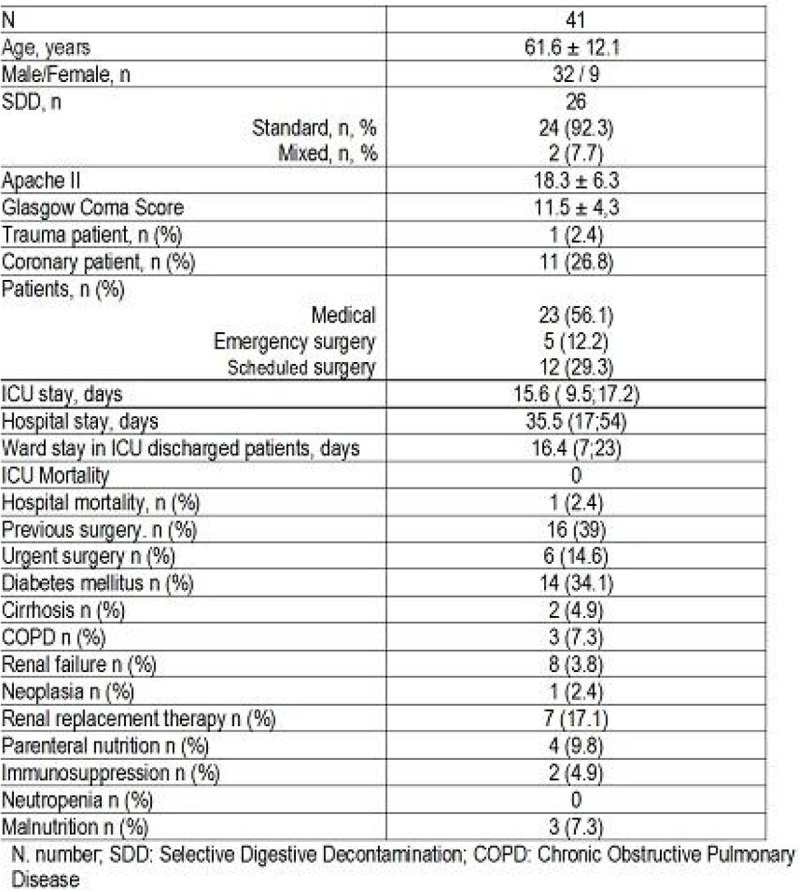
**Patients data**.

Forty one patients were analyzed, 26 of them received SDD (63,4%) and 24 of them (92.3%) received standard SDD. Demographic data, and admission types are shown in Figure 1

**Figure 2:**
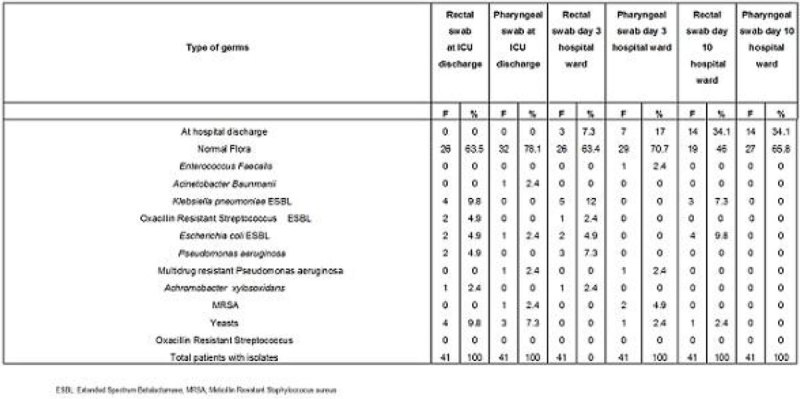
**Germs**.

Isolates with germs at ICU discharge and at hospital ward are shown in Figure 2.

The most frequent findings were negative isolates. Those who were positive at ICU discharge remained positive, and the negative ones remained negative except in 2 patients (one patient with a yeast at discharge changed to *Klebsiella pneumoniae*, and the other one changed from normal flora to *Pseudomonas aeruginosa*).There was only 1 patient who developed an infection in the ward originated in the UCI independently of receiving or not SDD. The patient was colonized by *Klebsiella pneumoniae* in the ICU and later developed a *Klebsiella pneumoniae* urinary infection in the hospital ward. The most frequent isolated germs at discharge and in the ward were *Pseudomona aeruginosa* and *Klebisella pneumoniae* (9%).

## Conclusions

All but two of patients the investigated patients receiving SDD in ICU did not have any change in the etiology of colonization after ICU discharge. Only another patient developed an attributable multi-resistant ICU infection.

